# Toxic Encephalopathy After Relapsed Indolent Non-Hodgkin’s Lymphoma Following Abrupt Discontinuation of Idelalisib (ZYDELIG®)

**DOI:** 10.7759/cureus.4043

**Published:** 2019-02-11

**Authors:** Mina Shenouda, Madhulika Urella, Salman Assad, Jennifer Dotson

**Affiliations:** 1 Oncology, Marshall University School of Medicine, Huntington, USA; 2 Internal Medicine, Marshall University School of Medicine, Huntington, USA

**Keywords:** oncology, chemotherapy

## Abstract

Idelalisib is a delta isoform-specific, phosphoinositide 3-kinase (PI3-K) inhibitor. It has been used as a single agent for the treatment of relapsed or refractory small lymphocytic lymphoma (SLL), follicular non-Hodgkin’s lymphoma (NHL), and in combination with rituximab for patients with chronic lymphocytic leukemia (CLL). We present a case of a 77-year-old man diagnosed with SLL and was treated with multiple chemotherapeutic regimens in the past. Considering multiple relapses, he was started on idelalisib monotherapy almost 12 months ago. The treatment was stopped due to worsening neutropenia as well as mixed response on the scans. Almost, within one week of stopping the medication, he presented with complaints of altered mental status, hematuria, and worsening of generalized lymphadenopathy. This time, the patient was started on venetoclax (BCL-2 inhibitor) and rituximab which he is tolerating well without any complications.

## Introduction

Idelalisib is one of the targeted therapies for refractory/relapsing small lymphocytic lymphoma (SLL). Idelalisib is an oral inhibitor of phosphoinositide 3-kinase (PI3K) delta that showed therapeutic activity in the initial studies of patients with non-Hodgkin's lymphoma (NHL). PI3K delta is integral to several signaling pathways in NHL cells including those that support survival proliferation and the retention of cells in lymphoid tissues. There are no clear guidelines for idelalisib discontinuation. Patients with a progression-free survival (PFS) less than the median expected for a treatment regimen are considered to have an early relapse. Abrupt discontinuation of idelalisib can also cause rapid disease progression resulting in complications [1].

## Case presentation

We present a 77-year-old male with a past medical history of NHL/SLL diagnosed almost 10 years ago, who presented to the hospital with abdominal swelling, altered mental status, and difficulty in urinating associated with hematuria. On physical examination, diffuse bulky lymphadenopathy was found in the cervical, axillary, and inguinal areas. Detailed oncologic history and treatment regimens that were taken by the patient have been well explained in Table 1.

**Table 1:** Oncologic history and treatment.

Timeline	Oncologic regimen
2008	Stage 4 non-Hodgkin's lymphoma (NHL)-small lymphocytic lymphoma (SLL) diagnosed with 11 weeks of fludarabine/rituxan.
2009	Positron emission tomography (PET) CT showed no evidence of disease and was started on maintenance rituxan therapy.
2010	First recurrence: left cervical worsening lymphadenopathy and was treated with bendamustine hydrochloride, bortezomib, rituxan x six cycles.
2011	Rituxan maintenance therapy.
2015	Second recurrence: rituxan, cyclophosphamide, doxorubicin, vincristine, and prednisone (R-CHOP) with partial response (PR) with later progression (PD).
2016	Diagnosed as refractory SLL. Started on idelalisib but disease progressed on this regimen based on imaging findings. Idelalisib was stopped and then started on ofatumumab. Initial dose: 300 mg on day 1, followed one week later by 2000 mg once weekly for seven doses (doses two to eight), followed four weeks later by 2000 mg once every four weeks for four doses. There was a relapse of disease <6 months with progressive lymphadenopathy after treatment with R-CHOP.
2017	Was refractory to ofatumumab and received chlorambucil/obinutuzumab for six cycles but progressed, and has been restarted on Idelalisib twice daily.
2018	Idelalisib therapy was stopped a week before pancytopenia and worsening abdominal swelling symptoms and started on venetoclax (B-cell lymphoma 2; BCL-2 inhibitor) 20 mg/day for seven days and 50 mg/day afterward.

**Figure 1:**
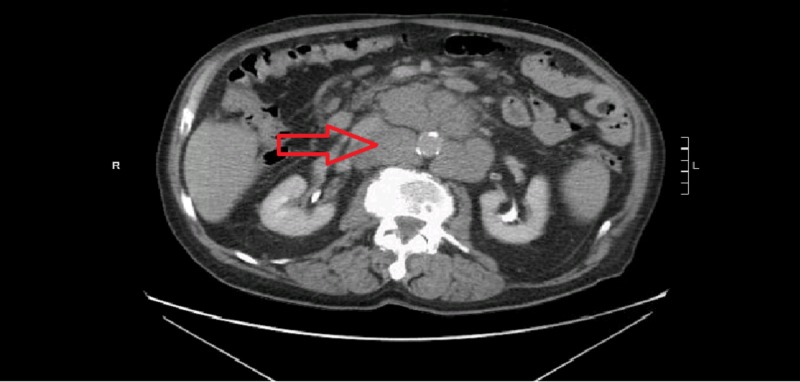
Computed tomography (CT) scan abdomen. Extensive paraaortic lymphadenopathy (red arrow).

**Figure 2:**
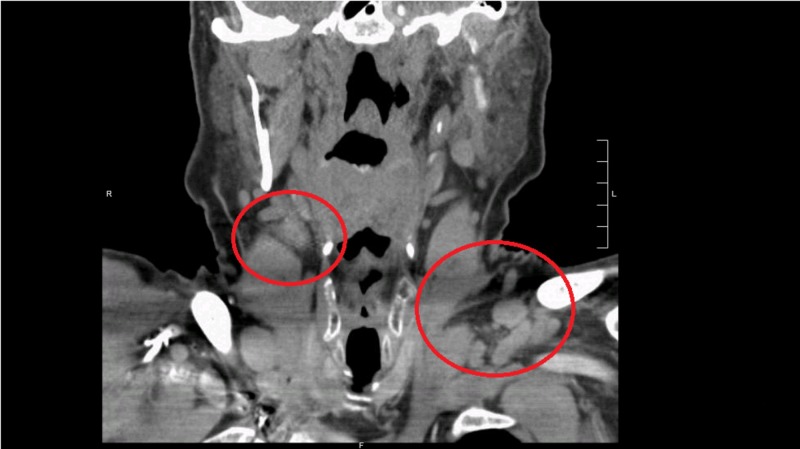
CT scan neck and soft tissue. Diffuse cervical lymphadenopathy along with enlarged clavicular lymph nodes (red circles).

**Figure 3:**
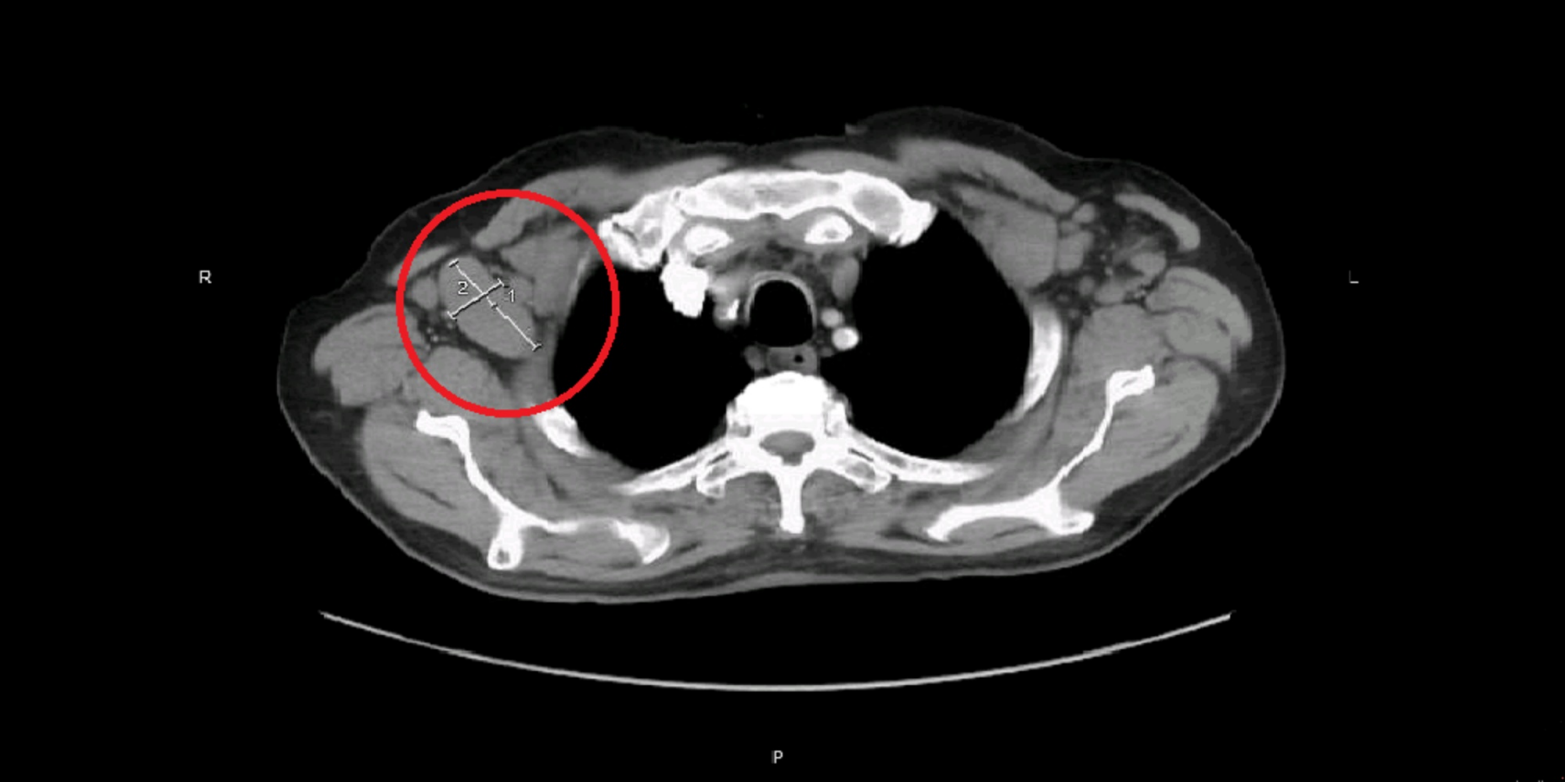
CT scan chest. Axillary lymphadenopathy (red circles).

Laboratory workup showed hypokalemia, hypophosphatemia, and elevated lactate dehydrogenase levels. During the hospitalization, computed tomography (CT) scan head was done that showed negative findings for any acute events. Due to altered mental status with underlying worsening of NHL and metabolic disturbances, the toxic and metabolic encephalopathy were the differentials under consideration. Metabolic derangements were corrected during hospitalization and that improved his mental status as well. Idelalisib treatment was discontinued abruptly a week prior to patient’s presentation to the hospital due to pancytopenia and a mixed response on the CT scan imaging. Upon admission, a repeat CT of the abdomen and pelvis showed diffuse bulky lymphadenopathy in the abdomen; one of the nodes in the anterior para-aortic region was measured about 5 cm × 5 cm × 8 cm (Figure 1). Bilateral iliac, inguinal, and retroperitoneal lymphadenopathy was also significantly increased in size compared with prior CT scan. There was diffuse lymphadenopathy along with axillary and cervical regions as well (Figures 2-3).

Bone marrow biopsy was done that showed a cluster of differentiation 5 (CD5) and CD23 positive B-cell population (37% of the lymphoid gate), lambda-restricted. Almost all of the B-cells showed immunophenotypic expression of CLL/SLL with lambda light chain restriction that was found in previous cases of this patient. Interestingly, a kappa light chain restricted population of monoclonal plasma cells co-expressed with CD56 (1.1% of total events) is also identified. Urology was consulted for urinary complaints of difficult voiding and hematuria. However, the patient was further diagnosed with paraphimosis and scheduled to have an elective circumcision that alleviated his urinary complaints later. Idelalisib treatment for SLL/NHL that patient took for almost one year was stopped a week prior to current clinical symptoms. Based on progressive SLL/NHL, the patient was started on venetoclax (B-cell lymphoma 2; BCL-2 inhibitor) 20 mg/day for seven days and 50 mg/day afterward. The patient did not develop any tumor lysis syndrome after starting the therapy and is tolerating the treatment well based on both imaging and clinical finding.

## Discussion

Idelalisib is an oral inhibitor of phosphoinositide 3-kinase (PI3K) delta (δ) that showed therapeutic activity in initial studies of patients with NHL. P13K delta is integral to several signaling pathways in NHL cells including those that support survival proliferation and the retention of cells in lymphoid tissues. Idelalisib, the first Food and Drug Administration (FDA)-approved PI3Kδ inhibitor, is a valuable and safe management option for patients with relapsed SLL or chronic lymphocytic leukemia (CLL) and relapsed follicular lymphoma. Based on progression-free survival (PFS) significance, idelalisib joins ibrutinib-Bruton's tyrosine kinase (BTK) inhibitor as a FDA-approved oral agent for use in patients with relapsed NHLs [2]. Acute transaminitis with elevations of alanine aminotransferase (ALT) or aspartate aminotransferase (AST) and neutropenia are the most common laboratory abnormalities from idelalisib therapy. A study also documented adverse effects (AEs) in patients treated with idelalisib including fever/pyrexia (10%), pneumonia (7%), and diarrhea (7%). These complications eventually led to treatment discontinuation in 20% patients and a dose reduction (either 100 mg twice a day or 75 mg twice a day) in 34% patients [3].

The patient in the present case showed a mixed response and developed neutropenia which prompted the oncologist to discontinue idelalisib therapy immediately. A subsequent CT of the abdomen showed significant worsening in the size of the lymph nodes leading to complications including intractable abdominal pain, and hematuria with possible ureteral obstruction within one week of idelalisib discontinuation. Afterward, instead of re-starting the same regimen of idelalisib, treatment was switched to venetoclax plus rituximab combination. O'Brien et al. documented that single-agent treatment with ibrutinib was associated with a five-year PFS rate of 92% in CLL/SLL treatment-naïve (TN) older patients (≥65 years of age) and 44% in relapsed/refractory (R/R) patients. The onset of cytopenias was also reduced over five-year follow up of patients with a single treatment of ibrutinib [4]. Barrientos et al. also recommended the same transition of therapy regimen in case of worsening of complications after discontinuation of idelalisib treatment [5]. Additionally, our Oncology team believed that tapered discontinuation of chemotherapy regimen would have avoided the complications of progression of the disease instead of abrupt withdrawal of treatment, and the outcomes would have been different as well. However, we will leave the platform for the discussion among the researchers, pharmacists, and physicians for the future reference and evaluation of better treatment control regimen.

## Conclusions

In summary, in a patient with refractory NHL being treated with idelalisib, we suggest performing the gradual discontinuation of idelalisib to prevent acute disease progression which may result in complications that can be life-threatening if not treated in a timely manner. A refractory case of SLL/NHL to multiple treatment regimens questions the understanding of underlying malignancy and also suggests evaluating alternate better treatment options.
